# Inhibition of CISD2 promotes ferroptosis through ferritinophagy-mediated ferritin turnover and regulation of p62–Keap1–NRF2 pathway

**DOI:** 10.1186/s11658-022-00383-z

**Published:** 2022-09-30

**Authors:** Yanchun Li, Bing Xu, Xueying Ren, Luyang Wang, Yaqing Xu, Yefeng Zhao, Chen Yang, Chen Yuan, Huanjuan Li, Xiangmin Tong, Ying Wang, Jing Du

**Affiliations:** 1 Department of Central Laboratory, Affiliated Hangzhou First People’s Hospital, Zhejiang University School of Medicine, Hangzhou, 310006 Zhejiang China; 2 Department of Clinical Laboratory, Laboratory Medicine Center, Zhejiang Provincial People’s Hospital (Affiliated People’s Hospital, Hangzhou Medical College), Hangzhou, 310014 Zhejiang China; 3 Department of Clinical Laboratory, Hangzhou Women’s Hospital, Hangzhou, 310016 Zhejiang China; 4 Department of Laboratory Medicine, The Second Affiliated Hospital of Zhejiang Chinese Medical University, 310005 Hangzhou, Zhejiang China

**Keywords:** CISD2, Iron, Autophagy, Ferroptosis, Ferritinophagy

## Abstract

**Background:**

CDGSH iron sulfur domain 2 (CISD2) is an iron–sulfur protein with a [2Fe–2S] cluster, which is critical for cell proliferation and iron homeostasis. It has been demonstrated that aberrant expression of CISD2 is associated with the progression of multiple cancers. However, the underlying mechanism of CISD2 in regulating tumorigenesis remains obscure.

**Methods:**

Bioinformatics strategies were used to investigate the protein interaction network and functional annotation of CISD2. In the functional experiment, cell viability was measured by CCK-8 kit. The levels of cellular reactive oxygen species (ROS), intracellular free iron, lipid peroxides, and lysosomal activity were determined by DCF-DA, RPA, C11-BODIPY, and cathepsin B staining, respectively. The glutathione (GSH) content was determined using a GSH assay kit.

**Results:**

We showed that knockdown of CISD2 significantly accelerated the Erastin-induced ferroptotic cell death with excess lipid peroxidation, GSH exhaustion, and iron accumulation, while overexpression of CISD2 hindered the sensitivity to Erastin. Further assays via confocal microscopy and western blot exhibited that CISD2 knockdown markedly enhanced the lysosomal activity, and activated ferritinophagy under the exposure of Erastin. Pharmacological inhibition of lysosomal function could inhibit the degradation of ferritin heavy chain (FTH), and attenuate the phenotypes of ferroptosis, such as accelerated iron accumulation and lipid peroxidation. Notably, we found that Erastin-induced compensatory elevation of nuclear factor erythroid 2-related factor 2 (NRF2) could be eliminated in CISD2 depletion cells. Mechanically, CISD2 knockdown promoted the degradation of autophagy adaptor p62 and resulted in an increased binding affinity of Keap1 with NRF2, thus leading to the increased ubiquitination and subsequent degradation of NRF2. Enforced expression of NRF2 reversed the sensitivity of shCISD2 cells to ferroptosis both in vitro and in vivo. Conversely, enforced expression of Keap1 exacerbated the degradation of NRF2, reduced the transcriptional expression of FTH and heme oxygenase 1 (HO-1), increased the oxidative damage, and thus further facilitated ferroptosis.

**Conclusion:**

Taken together, our current results illustrated two parallel mechanisms involved in the shCISD2-mediated ferroptosis. One was that shCISD2 enhanced the accumulation of free iron via ferritinophagy-dependent ferritin turnover; the other was that CISD2 depletion induced the inhibition of the p62–Keap1–NRF2 pathway, which resulted in oxidative stress and ferroptosis.

**Supplementary Information:**

The online version contains supplementary material available at 10.1186/s11658-022-00383-z.

## Introduction

Since the discovery of ferroptosis in 2012, this type of regulated cell death (RCD) attracts global attention for its crucial role in several pathophysiological conditions, including tumorigenesis, neurodegenerative diseases, and ischemia–reperfusion injury [1–3]. The specific process of ferroptosis is characterized by the catastrophic accumulation of free iron and unrestricted lipid peroxidation [4]. Several signal transduction pathways, including iron metabolism, GSH-GPX4, NRF2, and FSP1-COQ10, have been found involved in the regulation of ferroptosis [5, 6]. Additionally, we and others have previously discovered that ferroptosis is also an autophagic cell death process activated via a form of cargo-specific autophagy known as ferritinophagy, which depends on the degradation of ferritin to release free iron ions [7, 8]. This is a crosstalk between autophagy and ferroptosis through regulation of iron homeostasis. Increasing evidence have revealed that cancer cells do exhibit an enhanced dependence on iron relative to normal cells, a phenomenon termed iron addiction [9–11]. It is worth noting that highly soluble Fe^2+^ is a prerequisite for the generation of lipid peroxides and ferroptosis. Thus, inducing ferroptosis could act as a promising therapeutic strategy and eliminate the drug resistance of cancers [12]. Although the essential features of ferroptosis have been established, the specific molecular mechanisms underlying the regulatory network remain elusive. Therefore, more related proteins or regulators for ferroptosis should urgently be explored.

Iron–sulfur cluster is an ancient prosthetic group that is essential for biological processes such as iron homeostasis, redox homeostasis, heme synthesis, enzymatic function, and regulation of gene expression. Impaired iron–sulfur cluster assembly not only affects activities of various iron–sulfur enzymes, such as succinate dehydrogenase and aconitase, but also results in iron starvation stress with excess iron overload. Our recent study demonstrated that cancer cells depend on high levels of iron–sulfur cluster biosynthesis, and inhibition of frataxin, one of the iron–sulfur cluster biosynthetic enzymes, initiated the ferroptotic cell death through induction of free iron accumulation and mitochondrial dysfunction [13]. This is in line with the research by Alvarez showing that suppression of iron–sulfur assembly protein NFS1 makes cancer cells sensitive to ferroptosis and slows tumor growth [14]. These studies revealed that iron–sulfur cluster plays an important role in ferroptotic cell death.

The NEET protein family contains a group of highly conserved iron–sulfur proteins that harbor 3Cys-1His [2Fe–2S]-binding CDGSH domain, and play an important role in human health and disease [15]. They are unique for their redox-activated [2Fe–2S] cluster, which can easily be protonated [16, 17]. In humans, only three genes are currently known to encode NEET proteins. For example, *CISD1* codes for a homodimeric protein CISD1, also known as mitoNEET, that is localized to the outer mitochondrial membrane and participates in oxidative regulation. Besides, *CISD2* encodes for CISD2, also known as NAF-1, which is localized to the outer mitochondrial membrane and endoplasmic reticulum, and participates mainly in autophagy regulation, calcium/iron homeostasis, oxidative stress, and mammalian lifespan control [18, 19]. Moreover, CISD2, one of the most studied representatives of the NEET family, has been identified as being involved in a number of pathologies, including obesity, diabetes, aging, and neurodegeneration, and is rapidly gaining promise as a target for cancer therapy [20, 21].

In the current study, through a series of cellular, molecular, and pharmacological analyses, we demonstrated that the NEET protein CISD2 exerts a crucial role in the regulation of ferroptosis through maintaining iron homeostasis and redox equilibrium.

## Materials and methods

### Antibodies and reagents

The antibodies to CISD2 (60758S) and ATG7 (8558S) were obtained from Cell Signaling Technology (Danvers, MA); antibodies to NRF2 (ab62352), GPX4 (ab125066), FTH (ab75973), P62 (ab91526), p-P62 (ab211324), ATG5 (ab108327), Keap1 (ab227828), HO-1 (ab68477), P53 (ab179477), and DCF-DA were obtained from Abcam (Cambridge, MA); antibody to LC3 (L7543) was obtained from Sigma-Aldrich (St. Louis, USA); Cell Counting Kit-8 (CCK-8) Assay Kit and Erastin were obtained from Meilunbio (Dalian, China); C11-BODIPY (581/591), deferoxamine (DFO), Z-VAD-FMK, BafA1, *N*-acetylcysteine (NAC), GSH, ferrostatin-1, and necrosuifonamide (Necro) were obtained from Selleck Chemicals (Houston, TX); Lip3000 Transfection Reagent and BCA Protein Assay Kit were obtained from Thermo Fisher Scientific (Waltham, MA); GAPDH antibody, Ad-GFP-LC3B, and GSH Assay Kit were purchased from Beyotime (Shanghai, China). Magic Red cathepsin B (Immunochemistry Technologies) was obtained from Immunochemistry Technologies (Bloomington, USA) to assess the activity of cathepsin B.

### Cell culture

293T (SCSP-502) and HT1080 (TCHu170) cell lines were obtained from the Chinese Academy of Sciences (Shanghai, China) and cultured in DMEM (Hyclone, Logan, UT, USA) supplemented with 10% fetal bovine serum (FBS; Gibco, Grand Island, NY, USA), 100 U/mL penicillin, and 100 mg/mL streptomycin (Beyotime, Shanghai, China). HL60 (TCHu 23) cell line was obtained from the Chinese Academy of Sciences (Shanghai, China) and cultured in RPMI-1640 (Hyclone, Logan, UT, USA) supplemented with 10% FBS. Cells were maintained in a humidified incubator containing 5% CO_2_ at 37 ℃.

### Enrichment analysis of CISD2-related genes

STRING (https://www.string-db.org/) website was used to investigate the CISD2-related genes according to the routine parameters [organism: *Homo sapiens*; network type: full STRING network; required score: low confidence (0.150); network edges: confidence and max number of interactors (100) in first shell]. Then, a gene list containing the top 100 CISD2 binding proteins was obtained. To conduct an intensive analysis, we uploaded the gene list into Metascape (https://metascape.org/) under certain conditions (input and analysis species: *H. sapiens*), and the available enrichment analysis results and functional protein association networks were derived. Moreover, DAVID (http://david.ncifcrf.gov) database was used to perform the functional annotation of CISD2 for Gene Ontology (GO) and Kyoto Encyclopedia of Genes and Genomes (KEGG) pathway analysis. The data of GO, containing biological process (BP), cell component (CC), and molecular function (MF), and KEGG were visualized by bubble plots using ggplot 2R packages.

### Construction of lentiviral vectors

Full-length cDNAs of FTH, Keap1, and NRF2 were ordered from Sino Biological (Beijing, China), and subcloned into pLVX-IRES-Neo lentivirus vector (Takara, Dalian, China) by Seamless Cloning kit (TransGen Biotech, Beijing, China). The recombinant vectors were verified by gene sequencing.

### Lentivirus packaging

293T cells were used for lentivirus packaging. Briefly, the recombinant lentivirus vector were co-transfected with pMD2G and pSPAX2into 293T cells according to the instructions of Lipo3000 transfection kit. After 48 h, the supernatant was collected and filtered by 0.45 μm filter and stored at −80 ℃ for usage.

### Construction of stable cell lines with gene overexpression

Cells were seeded in 12-well plate and transfected with the packaged lentivirus, respectively. After 12 h, the cells were cultured in fresh complete culture medium for another 24 h. Then selective antibiotics were used for screening for 7 days. Western blot was conducted to assess the expression of modified genes. The verified cells were preserved for subsequent experiments.

### Cell viability assay

Cells (1 × 10^4^ cells per well) were seeded in 96-well plates (Nest Biotechnology) and cultured with the indicated concentration of chemicals for 12 h. At the end of treatment, 10 µL CCK-8 reagent was added to each well for 2 h incubation at 37 ℃. The absorbance value at 450 nm was measured by microplate reader to evaluate the cell viability. Each group was performed in triplicate.

### Protein extraction and western blot

After indicated treatments, cells were harvested and washed twice with ice-cold phosphate-buffered saline (PBS). Then, the cell pellets were lysed in a cooling RIPA lysis buffer (Beyotime, P0013B) containing complete protease and phosphatase inhibitors (Thermo Fisher Scientific, Waltham, MA) for 15 min and centrifuged at 18,000 rpm for 10 min. The concentration of proteins in supernatant was determined by the BCA protein assay kit (Thermo Fisher Scientific, Waltham, MA). Equal amount of samples was boiled in SDS-loading buffer at 95 ℃ for 10 min, and separated by SDS-PAGE. Next, the separated proteins were transferred onto the polyvinylidene fluoride (PVDF) membranes, which were then blocked with 5% fat-free milk for 1 h at room temperature and incubated with primary antibodies overnight at 4 ℃. After being washed with Tris buffer saline containing 0.1% Tween-20 (TBST) three times, the protein bands were incubated with secondary antibodies for 1 h at room temperature and washed with TBST three times again. The intensity of protein expression was visualized by an enhanced chemiluminescence system.

### Real-time PCR

The total RNAs from CISD2 knockdown HT1080 cells with or without Erastin treatment were extracted by the AG RNAex Pro Reagent (Accurate Biology). Afterward, RNAs were reversed into cDNAs through Evo M-MLV RT Kit (Accurate Biology) according to the manufacturer’s instructions. The cDNAs were used as templates for the quantification of NQO1 expression by the SYBR green chemistry detection method in RT-PCR reactions. The gene expression level of GAPDH was used for normalization. The nucleotide sequences of primers were listed as follows: NQO1, forward, 5′-CCTGCCATTCTGAAAGGCTGGT, reverse, 5′-GTGGTGATGGAAAGCACTGCCT; GAPDH, forward, 5′-GCACCGTCAAGGCTGAGAAC, reverse, 5′-ATGGTGGTGAAGACGCCAGT.

### Cellular ROS detection

The oxidative-sensitive fluorescent probe DCF-DA was used to measure the generation of intracellular ROS. Briefly, after indicated treatments, cells were washed twice with PBS and incubated with 5 µM DCF-DA for 30 min at 37 ℃ in the dark. Equal amount of cells was washed and subsequently suspended in culture medium for microplate reader analysis. Green fluorescence intensity was positively correlated with level of ROS.

### GSH analysis

The treated cells were harvested, washed twice in PBS, and lysed in RIPA lysis buffer. Then, the GSH content was determined using a commercially available GSH assay kit (Beyotime, S0053) following the manufacturers’ protocol.

### Detection of cellular iron

The nontoxic iron-sensitive fluorescent probe RPA was used to measure the level of intracellular free iron. Briefly, after indicated treatments, cells were washed twice with PBS and incubated with 4 µM RPA for 20 min at 37 ℃ in the dark. The cells were washed three times again and subsequently suspended in culture medium for flow cytometry analysis. The intensity of red fluorescence was negatively correlated with the level of free iron.

### Detection of lipid peroxides

Lipid peroxides were examined by C11-BODIPY 581/591 (BODIPY) staining. Briefly, at the end of treatment, cells were incubated with BODIPY at a final concentration of 5 µM for 30 min at 37 ℃ in the dark. Then, the washed cells were viewed and photographed by confocal microscope, or analyzed via flow cytometry to determine the intensity of green fluorescence, which was positively correlated with the level of lipid peroxides.

### Detection of the formation of LC3 puncta

Control and CISD2-silenced cells transfected with LC3-GFP adenovirus were seeded in the confocal chamber and treated with or without Erastin for 8 h. Then cells were washed with PBS three times and incubated with fresh culture medium for observation under a confocal microscope. The formation of punctate green fluorescence (LC3 puncta) within the cell was counted.

### Lysosomal activity assay

At the end of treatments, cells were stained with Magic Red cathepsin B probe for 90 min at 37 ℃ in the dark, then washed with PBS and visualized under a confocal microscope. The intensity of red fluorescence was positively correlated with the activity of cathepsin B.

### Xenograft models

All animal experiments were approved by the ethics committee of Zhejiang Provincial People’s Hospital. Six-week-old male BALB/c nude mice were purchased from Jiangsu Jizui Yao Kang Biological Technology Co. LTD. CISD2-silenced HT1080 cells (4 × 10^6^) with or without NRF2 overexpression were subcutaneously injected into the flank. Once the tumors reached approximately 100–200 mm^3^, the mice were randomly divided into two groups with the treatment of vehicle or Erastin, respectively. At the end of the experiments, the mice were euthanized by CO_2_ inhalation (with a flow rate 20% per minute) and the tumors were dissected and photographed, following the detection of GSH content.

### Analysis of the iron and malondialdehyde (MDA) content in tumor tissues

For the detection of iron and MDA content, protein samples were firstly extracted and isolated from the tumor tissues, and qualified by a Pierce BCA protein detection kit (Thermo Scientific, 23227). The iron contents in these protein samples were determined using ferroZine as described previously [22]. The level of MDA was detected by an MDA assay kit (Solarbio, China). Briefly, equal amounts of protein samples were incubated with the reaction buffer for 60 min at 100 ℃. After being cooled on ice, the mixtures were centrifuged for 10 min at 10,000*g*. Then, 200 µL supernatant of each sample was used to determine the absorbance at 532 nm. The absorbance at 600 nm was measured at the same time as the baseline, and the relative MDA content of each sample was statistically analyzed.

### Statistical analysis

All data are shown as mean ± standard deviation (SD) from at least three independent experiments. The differences between two groups were analyzed by Student’s *t*-test, and the differences among three or more groups were analyzed by two-way analysis of variance (ANOVA). Statistical analysis was performed using Graphpad Prism 6.0, and *P *< 0.05 was considered to be statistically significant.

## Results

### Enrichment analysis of CISD2-related genes in KEGG pathway and GO terms

To gain a general understanding of CISD2 in cellular functions, we conducted big data analytics through STRING database (https://www.string-db.org/), and obtained a protein–protein interaction (PPI) network (Additional file 1: Fig. S1A), from which we selected 100 hub genes related to CISD2 for functional annotation. Regarding the GO terms of biological processes (BP), these genes were significantly enriched in autophagy and apoptotic process, macroautophagy, mitochondrial complex I assembly, iron–sulfur cluster assembly, and cellular iron homeostasis. Data from DAVID database showed that the associated KEGG pathways were mainly enriched in Parkinson’s disease, oxidative phosphorylation, oocyte meiosis, nonalcoholic fatty liver disease, metabolic pathways, Huntington’s disease, and so on. In terms of cellular component (CC), the analysis revealed significant enrichment in mitochondria and endoplasmic reticulum. For molecular function (MF), the involved pathways were voltage-gated anion channel activity, protein binding, iron–sulfur cluster binding, and so on (Fig. S1B). These results from public databases well confirmed the point that CISD2 plays a crucial role in iron homeostasis, mitochondrial function, autophagy, and the occurrence of disease.

### The expression of CISD2 is associated with Erastin-induced ferroptosis

To determine the role of CISD2 in ferroptosis, we constructed CISD2 knockdown and overexpression cell lines through lentivirus infection, and the expression of CISD2 was verified by western blot (Additional file 2: Fig. S2A–D). Then, cell survival was monitored in these cells under the challenge of ferroptosis inducer Erastin. Results showed that CISD2 knockdown caused evident cell death in both HT-1080 or HL60 cells (Additional file 2: Fig. S2E, G), whereas CISD2 overexpression rendered cells more resistant to Erastin-induced ferroptotic cell death (Additional file 2: Fig. S2F, H). Collectively, these findings supported the involvement of CISD2 in the regulation of ferroptosis.

### CISD2 knockdown accelerates Erastin-induced lipid peroxidation, GSH exhaustion, and iron accumulation

**Fig. 1:**
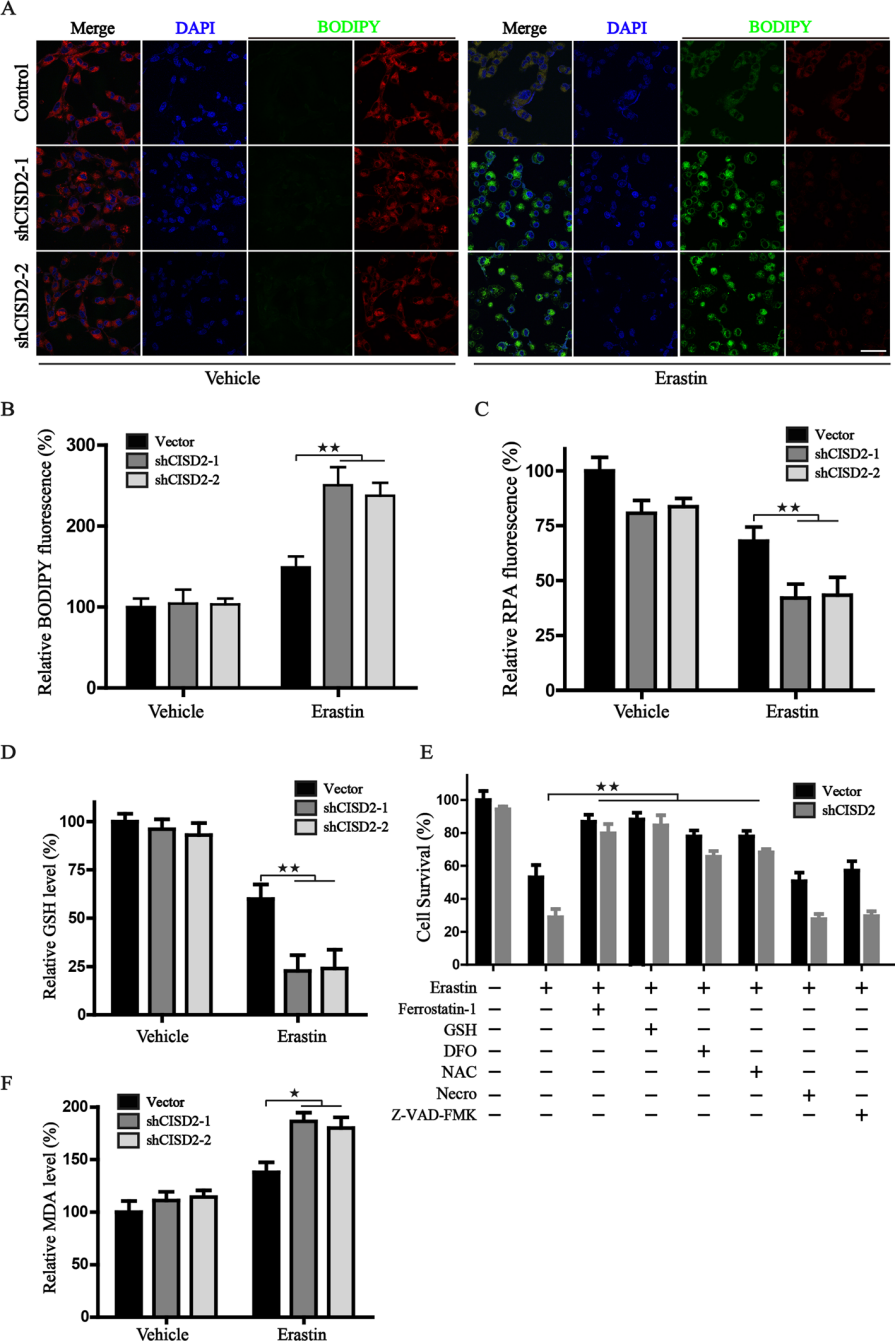
CISD2 knockdown accelerates the ferroptotic cell death induced by Erastin. **A** Detection of lipid peroxides in CISD2 modified HT-1080 cells through BODIPY staining and confocal microscope. The fluorescence images are representative of three independent experiments; the green fluorescence intensity was positively correlated with the level of lipid peroxides. Scale bar: 50 μm. **B** Statistical column graph of relative intensity of green fluorescence from BODIPY staining. **C** Detection of the cellular free iron by RPA staining. The relative intensity of RPA was calculated and the statistical graph was obtained from three independent experiments. **D** Detection of the GSH content in control or CISD2-silenced cells with or without the treatment of Erastin. **E** Cell viability assay in control or CISD2-silenced HT1080 cells after the treatment with Erastin (7.5 µM) alone or together with the indicated chemicals (1 µM ferrostatin-1, 0.5 mM GSH, 100 µM DFO, 0.5 mM NAC, 0.5 µM Necro, 5 µM Z-VAD-FMK), the cell viability in control cells treated with vehicle was defined as 100%, relative cell viabilities were calculated, and statistical graph was obtained from three independent experiments. **F** Detection of lipid peroxidation by MDA assay kit; the relative MDA content was calculated. ^★^*P* < 0.05, ^★★^*P* < 0.01 between indicated groups

It is well known that ferroptosis is characterized by iron-dependent unrestricted lipid peroxidation. We detected the ferroptotic events by the following assays and found (i) inhibition of *CISD2* increased the generation of lipid peroxides, as evidenced by the increased green fluorescence intensity of BODIPY staining under Erastin treatment (Fig. 1A, B); (ii) CISD2 suppression robustly activated the labile iron level characterized by the decreased fluorescence of RPA after Erastin treatment (Fig. 1C); (iii) cellular GSH content rapidly exhausted in shCISD2 cells subjected to Erastin (Fig. 1D); (iv) the content of MDA, an indicator of lipid peroxidation, increased obviously in shCISD2 cells under the challenge of Erastin (Fig. 1F). Additionally, several inhibitors of cell death were used to further characterize the mechanism of CISD2 knockdown-mediated cell death. Along with CISD2 silencing, the ferroptosis inhibitor (ferrostatin-1), iron chelator (DFO), and antioxidant (GSH, NAC) were capable of alleviating the impaired cell viability induced by Erastin incubation, while the inhibitors of apoptosis (Z-VAD-FMK) and necroptosis (necrosulfonamide) displayed no obvious recovery in terms of viability in CISD2 depletion cells (Fig. 1E). Collectively, these findings demonstrated that inhibition of *CISD2* promotes ferroptotic events with accelerated lipid peroxidation, excess free iron accumulation, and exhaustion of GSH.

### CISD2 knockdown activates ferritinophagy-dependent ferritin turnover

**Fig. 2:**
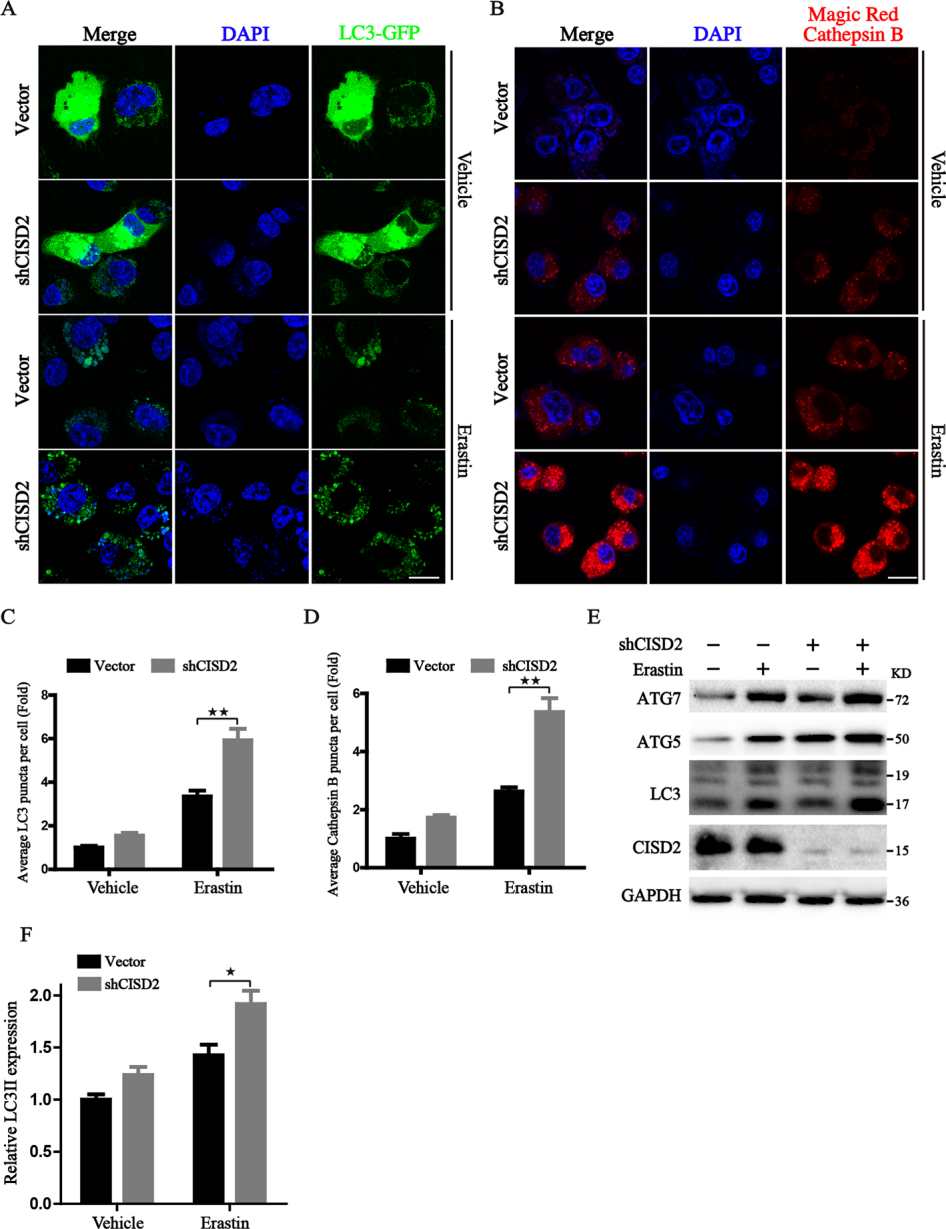
CISD2 knockdown promotes the activation of autophagy. **A** Representative images of the LC3 punches formation by confocal microscope in HT-1080 cells transfected with LC3-GFP adenovirus with or without Erastin (7.5 µM) treatment for 2 h. Scale bar: 10 μm. **B** Detection of the activity of lysosome by Magic Red cathepsin B staining and confocal microscopy. The intensity of red fluorescence was positively correlated with the lysosomal activity. Scale bar: 10 μm. **C**, **D** Statistical graph of LC3-GFP and cathepsin B staining, respectively (*n* = 3). **E** Western blot analysis of the expression of proteins associated with autophagy; GAPDH was used as loading control. **F** Quantitative analysis of LC3 expression; ^★★^*P* < 0.01 between the indicated groups

**Fig. 3:**
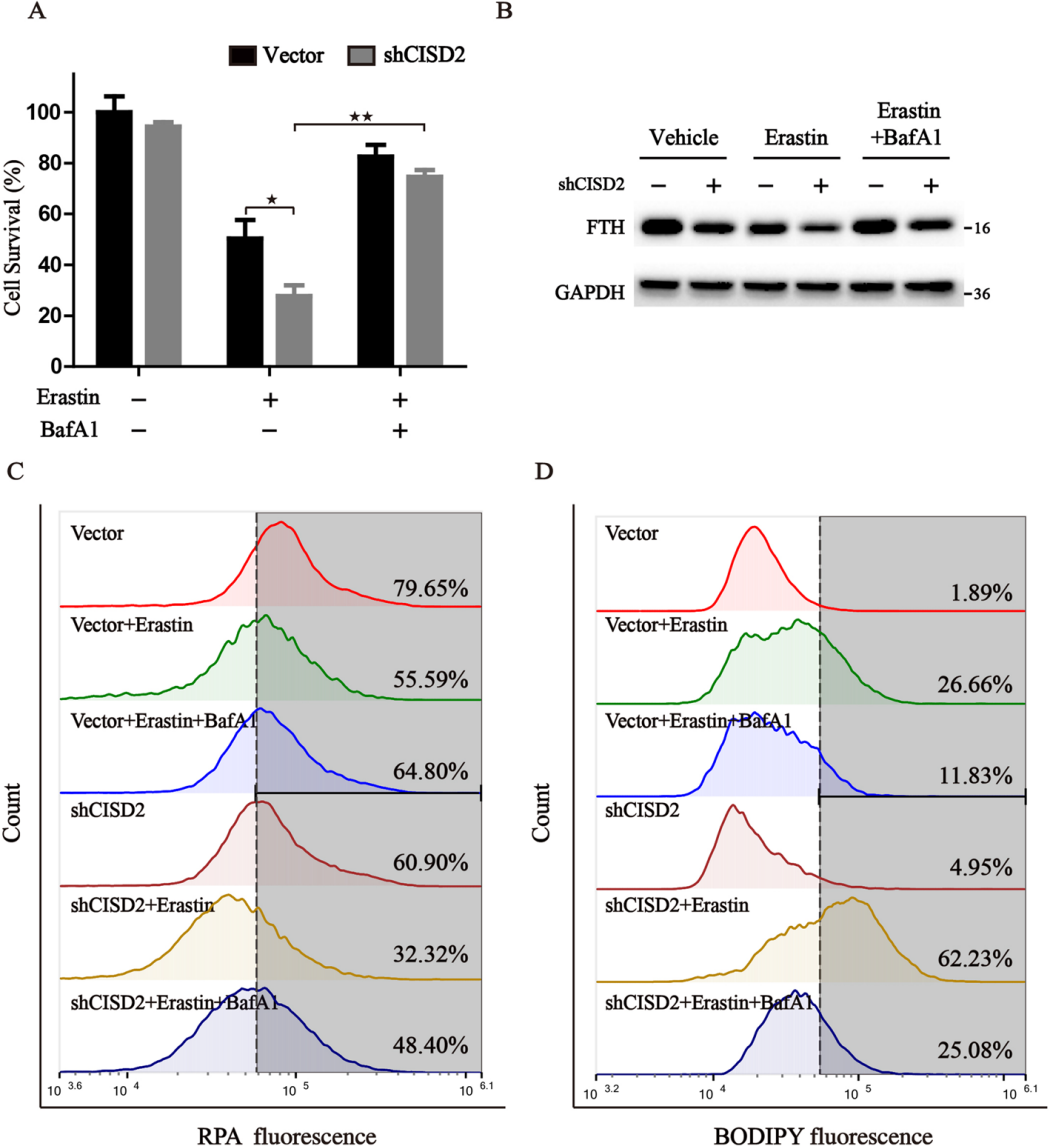
Inhibition of autophagy hinders the process of ferritinophagy and ferroptosis induced by CISD2 knockdown. **A** Cell survival assay in control or CISD2-silenced HT-1080 cells under Erastin treatment (7.5 µM) with or without BafA1 (40 nM) co-treatment. **B** Western blot analysis of FTH expression on the condition of autophagy inhibition; GAPDH was used as loading control. **C**, **D** Representative images from flow cytometry for the detection of free iron and lipid peroxides by the staining of RPA and BODIPY, respectively; the intensity of RPA fluorescence was negatively correlated with iron accumulation, and the intensity of BODIPY fluorescence was positively correlated with lipid peroxides. ^★^*P* < 0.05 and ^★★^*P* < 0.01 between the indicated groups

Ferroptosis is an autophagic cell death process promoted by the degradation of ferritin through the ferritinophagy process. We first transfected HT-1080 cells with GFP-LC3 protein, which is engaged in the expansion of autophagosomal membranes and considered an indicator for autophagosome. We found that Erastin significantly changed the distribution pattern of GFP-LC3 from diffused form into clustered puncta (Fig. 2A), and the average numbers of GFP-LC3 puncta were markedly increased in the CISD2-depletion cells under the exposure of Erastin (Fig. 2C). To confirm whether CISD2 knockdown accelerated the ferritinophagy process, we investigated lysosomal function by detecting cathepsin enzyme activities via Magic Red fluorescence probe. Confocal assay revealed that knockdown of CISD2 dramatically decreased lysosomal pH and increased the number of cathepsin B vesicles (Fig. 2B, D). We also confirmed these phenomena by western blot assay, and the results showed that CISD2 knockdown upregulated the expression of ATG7 and ATG5, and induced the conversion of autophagy related protein LC3 from a soluble form (LC3-I) to a liposoluble form (LC3-II) (Fig. 2E, F). These observed data prompted us to further examine whether shCISD2-activated lysosomal function participates in the process of ferroptosis. Therefore, lysosome was pharmacologically inhibited by the utilization of BafA1, and the results from cell survival assay demonstrated that BafA1 could restore ferroptotic cell death in shCISD2 cells to a level similar to that in the control cells (Fig. 3A). Notably, we found that BafA1 attenuated the ferritinophagy process in shCISD2 cells through the following assays: (i) BafA1 inhibited the degradation of ferritin in shCISD2 cells (Fig. 3B, Additional file 3: Fig. S3); (ii) BafA1 was able to block the accumulation of free iron in shCISD2 cells (Fig. 3C); (iii) BafA1 exhibited a strong effect on the alleviation of the lipid peroxidation in shCISD2 cells (Fig. 3D). In summary, these data indicated that CISD2 knockdown activates the function of lysosome, and provided definitive evidence linking CISD2 with ferritinophagy-mediated ferroptosis.

### Autophagy-dependent inhibition of NRF2 contributes to another mechanism involved in shCISD2-mediated ferroptosis

**Fig. 4:**
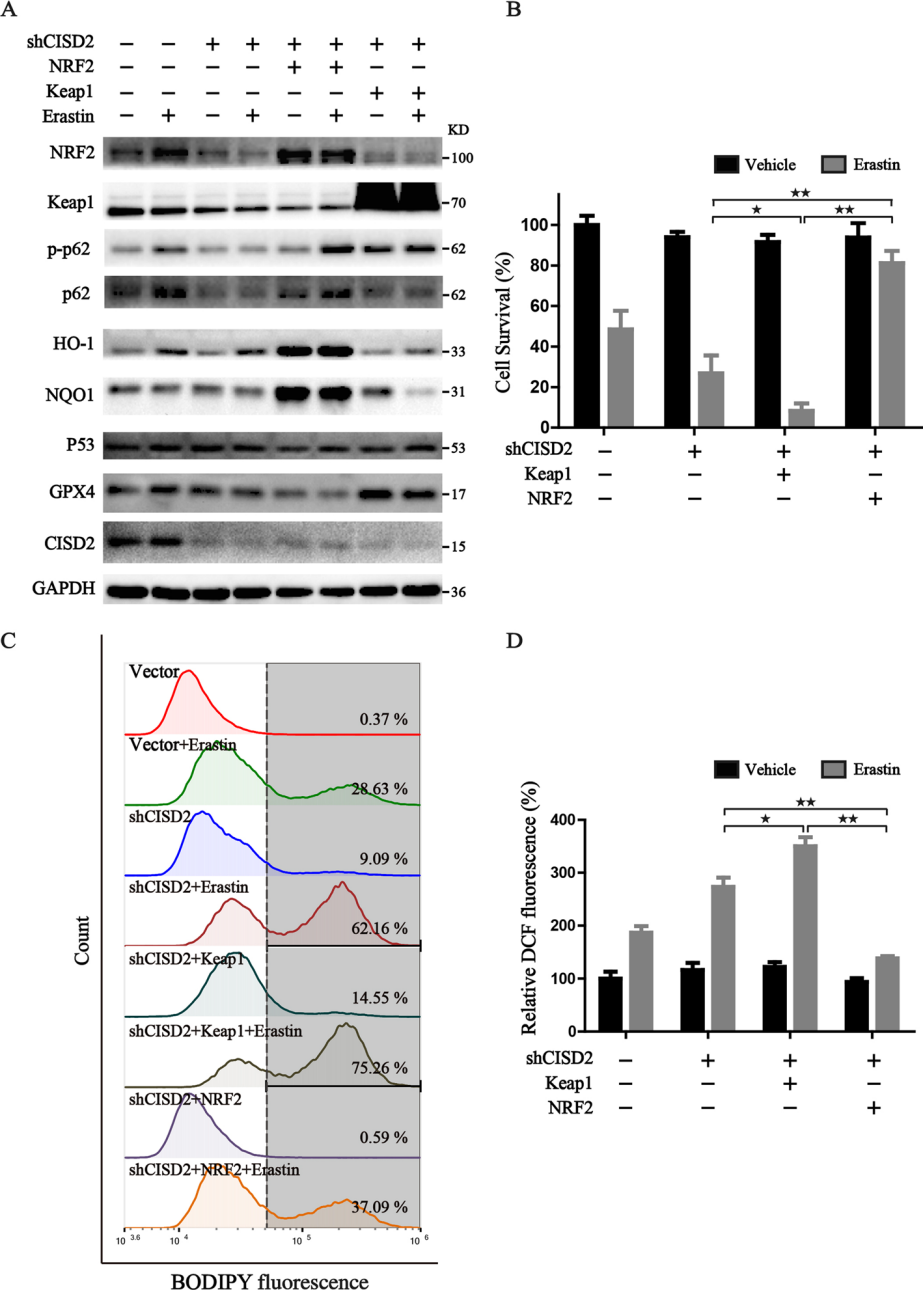
The p62–Keap1–NRF2 pathway is involved in shCISD2-mediated ferroptosis. **A** Western blot analysis of the expression of proteins associated with the p62–Keap1–NRF2 pathway and ferroptosis in HT-1080 cells; GAPDH was used as loading control. **B** Cell viability assay in the indicated cells with or without Erastin treatment; cell viability in control cells was defined as 100%. **C** Representative images from flow cytometry for the detection of lipid peroxides by BODIPY staining in the indicated HT-1080 cells with or without Erastin treatment (7.5 µM). **D** Statistical graph of DCF fluorescence from microplate reader to detect the cellular ROS in the indicated HT-1080 cells with or without Erastin treatment (7.5 µM); ^★^*P* < 0.05 and ^★★^*P* < 0.01 between the indicated groups

A previous study by Tang et al. elucidated that the p62–Keap1–NRF2 pathway is compensatorily activated in parallel with the occurrence of ferroptosis to protect cancer cells against ferroptosis [23]. We then wondered whether the activated autophagy flux induced by CISD2 knockdown could accelerate the ferroptotic cell death via downregulating the p62–Keap1–NRF2 pathway. Indeed, we found a similar phenomenon that Erastin induced a compensatory elevation of NRF2, whereas CISD2 depletion eliminated the adaptive response, and further facilitated the ferroptotic cell death (Fig. 4A, B). In addition, the transcriptional activity of NRF2 was also attenuated in CISD2 knockdown cells (Fig. S4A). Mechanistically, western blot assay confirmed that CISD2 knockdown promoted the degradation of autophagy adaptor p62. Meanwhile, decreased phosphorylation of p62 was identified under Erastin treatment in shCISD2 cells (Fig. 4A), which markedly reduced its binding affinity to Keap1, and led to the increased degradation of NRF2 through binding with Keap1 [24]. Importantly, we did not observe an obvious expression change of other ferroptotic regulators, such as p53 and GPX4 (Fig. 4A), demonstrating that these pathways were not the key events involved in the mechanism of shCISD2-initiated ferroptosis. To further explore whether the p62–Keap1–NRF2 pathway participated in the ferroptosis in shCISD2 cells, exogenous NRF2 or Keap1 overexpression cell lines were obtained through lentivirus transfection in CISD2-silenced cells. Western blot assay was utilized to examine the transfection efficiency (Fig. 4A). Moreover, overexpression of NRF2 largely inhibited lipid peroxidation, ameliorated cellular oxidative stress, and rescued the ferroptosis in CISD2-silenced cells (Fig. 4B–D). Conversely, enforced expression of Keap1 exacerbated the degradation of NRF2, reduced the transcriptional expression of NQO1, increased the oxidative damage, and thus further facilitated ferroptosis (Fig. 4B–D, Additional file 4: Fig. S4B). Overall, these findings manifested that the inhibition of NRF2 through autophagy-dependent p62 degradation plays a key role in the mechanism of shCISD2-initiated ferroptosis.

### NRF2 overexpression promotes the resistance of shCISD2 cells to ferroptosis in vivo

**Fig. 5:**
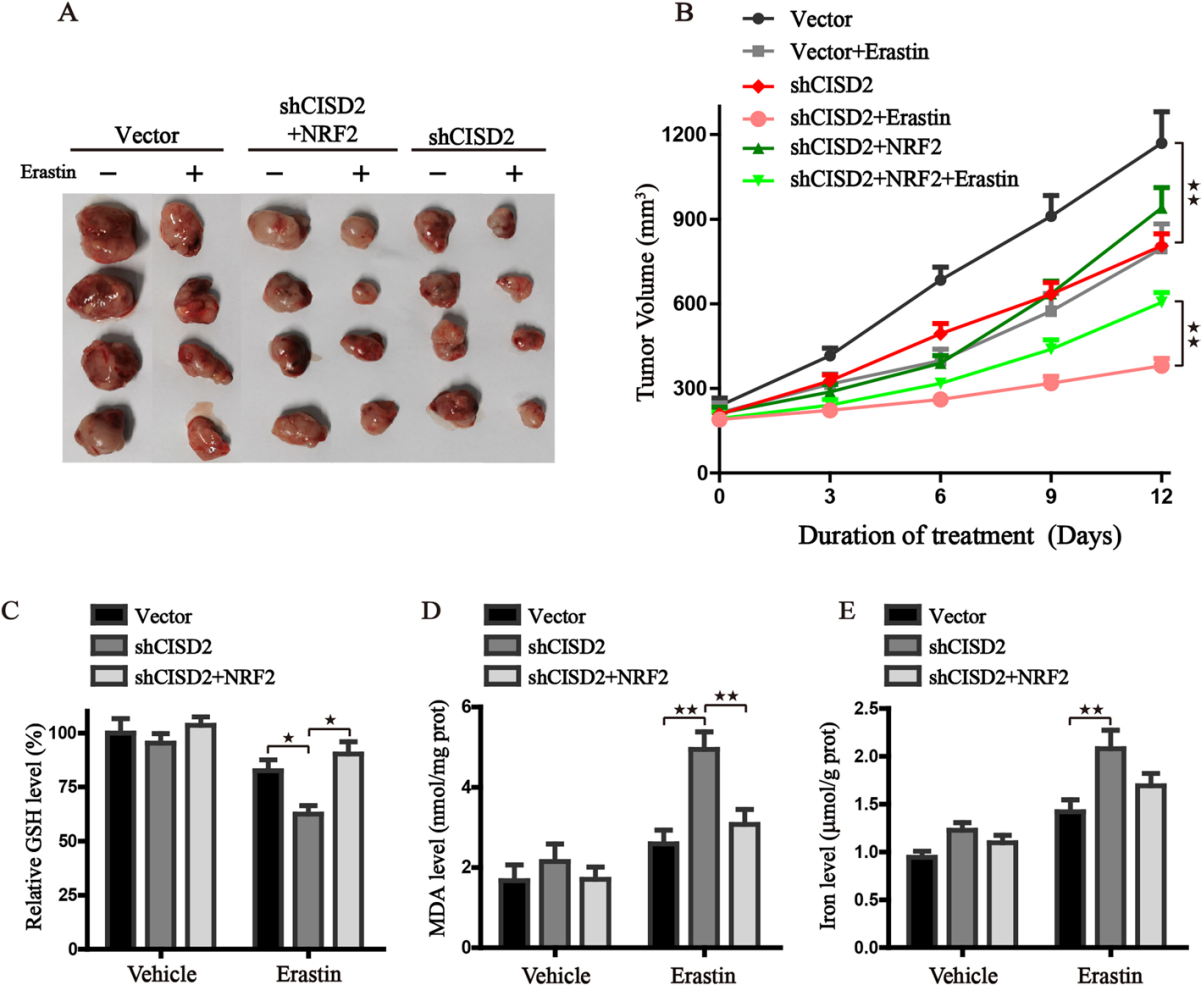
NRF2 overexpression promotes the growth of xenografts via enhancing antioxidation. **A** The gross morphology of subcutaneous tumors in nude mice of each indicated group. **B** The curves of time-dependent tumor volume after the injection of Erastin. **C**–**E** Detection of the GSH, MDA, and iron content, respectively, in tumor tissues from each group; protein concentration was used for calibration. ^★^*P* < 0.05 and ^★★^*P* < 0.01 between the indicated groups

The subcutaneous tumor models bearing gene-modified HT-1080 cells were also employed to further investigate whether the axis of NRF2 played a regulatory role in the shCISD2-mediated ferroptosis. Mirroring our in vitro data, the in vivo data further demonstrated that CISD2 knockdown inhibited tumor growth in vivo, while enforced expression of NRF2 reversed the sensitivity of shCISD2 cell to ferroptosis and boosted the size of tumors (Fig. 5A, B). Additionally, the content of GSH in tumor tissues was also detected, and the results were in line with the in vitro points that CISD2 inhibition promoted the exhaustion of GSH, which could be regulated by the axis of the NRF2 (Fig. 5C). Furthermore, the lipid peroxidation and iron content of the tumor tissues were also detected. Results were consistent with the in vitro experiments showing that CISD2 knockdown accelerated the iron accumulation and lipid peroxidation (Fig. 5D, E). Collectively, these results indicated that CISD2 knockdown induces ferroptosis partly through the NRF2 pathway, which could serve as a promising therapeutic target, predisposing cancer cells to an increased risk of ferroptotic cell death.

## Discussion

Induction of different regulated cell death (RCD) processes, including ferroptosis, necroptosis, autophagic cell death, pyroptosis, and apoptosis, has emerged as a powerful strategy for the treatment of multiple malignancies [25]. Each RCD process occurs through individual subroutines and could be modulated by unique signal transduction pathways. Thus, the discovery of specific molecular mechanisms underlying their regulatory network holds great promise for the identification of therapeutic targets and provides insights into new therapeutic avenues in cancers. Since the discovery of ferroptosis in 2012, plenty of studies have elucidated that ferroptosis is initiated by the abnormal accumulation of lipid hydroperoxides resulting from the overload of soluble iron (Fe^2+^) [26, 27]. Iron is the most abundant and essential metallic element in living organisms on Earth. The highly reductive and soluble Fe^2+^ is incorporated into Fe–S clusters or heme and serves as catalysts and cofactors that participate in many biological processes, including cellular respiration, electron transport, cell proliferation, and gene expression, and constitute the labile free-iron pool. Alteration of free iron level or iron metabolism proteins contributes to the change of sensitivity to ferroptosis [28]. Therefore, triggering ferroptosis in iron-rich tumors (such as HCC [23], PDAC [29], NSCLC [30], and breast cancer [31]) may be promising to provide insights into new therapeutic avenues or reverse drug resistance in cancers. In our study, we identified that CISD2 knockdown accelerated the accumulation of free iron, and increased cellular ROS and lipid peroxidation, thus inducing the occurrence of ferroptosis in cancer cells. These results were identical to other research. However, in addition to iron metabolism, CISD2 involves many biological pathways (Additional file 1: Fig. S1), and the associated mechanism of antineoplastic activity may also be intricate.

NEET proteins are a novel class of [2Fe–2S] cluster-containing proteins that are localized to the outer mitochondrial, mitochondria-associated membranes, and endoplasmic reticulum (ER) [32]. In humans, the loss of CISD2 function is responsible for the neurodegenerative disorder Wolfram syndrome 2 (WFS2), which is characterized by premature aging [21]. CISD2 and mitoNEET are two representative NEET proteins with similar structural and functional characteristics and could transfer their [2Fe–2S] to the iron–sulfur cluster assembly protein FXN [17]. MitoNEET located in mitochondrial outer membrane was identified to function as an exporter of iron–sulfur clusters or iron between the cytoplasm and the mitochondria. CISD2 expression could protect the liver from oxidative stress, reduce the occurrence of mitochondrial DNA deletions, and protect the liver from the nonalcoholic fatty liver disease [33]. Sohn et al. demonstrated that the NEET proteins were central to human breast cancer cells, and rendered them resistant to oxidative stress by mediating iron and ROS homeostasis in mitochondria [34]. Other studies have reported that high expression of NEET proteins was associated with aggressive malignancies and poor clinical outcomes of various human cancers, whereas silencing its expression inhibited tumor proliferation [35–37]. For unique and labile [2Fe–2S] clusters, it is generally accepted that mitoNEET and CISD2 could represent a regulatory link between the maintenance of high iron and ROS in cancer cells, which have been reported as key mechanisms mediating the resistance to ferroptosis [38, 39]. Although a vast amount of information has been gathered on NEET proteins in the past few years, key questions related to the function of NEET proteins still need further study.

In this work, we found that CISD2 knockdown facilitated Erastin-induced ferroptosis in different cell types, accompanied with key ferroptotic events including accelerated ROS production and lipid peroxidation. On the contrary, enforced expression of CISD2 increased the resistance to ferroptotic cell death. We observed a similar phenomenon that CISD2 knockdown accelerated the free iron accumulation. DFO administration ameliorated free iron accumulation and Erastin-induced ferroptosis. Most importantly, CISD2 is localized in ER- and mitochondria-associated membranes, which is distinct from other NEET proteins, reflecting that mitochondria homeostasis maintenance may not be the unique mechanism linked with ferroptosis. Indeed, we presented evidence to strengthen that CISD2 knockdown caused the activation of autophagy, which participated in the recycling process for the autophagic turnover of ferritin in lysosomes, leading to the accumulation of bioavailable intracellular labile iron.

Recent studies have found that ferroptosis represents a complex relationship with excessive autophagy and lysosome activity through various feedback loops, including NCOA4-facilitated ferritinophagy [40], STAT3-induced lysosomal membrane permeabilization, BECN1-mediated xc^−^ system inhibition, RAB7A-dependent lipophagy, and HSP90-associated chaperone-mediated autophagy [41]. Additionally, NRF2 is a key regulator of oxidative stress response and transcription factor that prevents ferroptosis [42, 43]. In this work, we found that Erastin treatment induced a compensatory elevation of NRF2 in parallel with the occurrence of ferroptosis to protect cancer cells against cell death. However, depletion of CISD2 eliminated the adaptive elevation of NRF2 and further facilitated ferroptotic cell death. Mechanistically, CISD2 knockdown promoted the degradation of autophagy adaptor p62 and decreased phosphorylation of p62, thus resulting in the increased binding affinity of Keap1 with NRF2. Overexpression of NRF2 largely inhibited lipid peroxidation, ameliorated cellular oxidative stress, and rescued the ferroptosis in CISD2-silenced cells. Conversely, enforced expression of Keap1 exacerbated the degradation of NRF2, reduced the transcriptional expression of NQO1, increased oxidative damage, and thus facilitated ferroptosis.

In conclusion, our current results illustrated two parallel mechanisms involved in shCISD2-mediated ferroptosis: shCISD2 enhanced the accumulation of free iron via ferritinophagy-dependent ferritin turnover, and CISD2 depletion induced the inhibition of the p62–Keap1–NRF2 pathway, resulting in oxidative stress and ferroptosis. Of note, this study uncovered the role of CISD2 in molecular biological characteristics of cancer and provided a potential target predisposing cancer cells to an increased risk of ferroptotic cell death.

## Supplementary Information

**Additional file 1: Figure S1.** Enrichment analysis of CISD2 related genes. (A) Protein-protein interaction network of CISD2 from STRING website (https://www.string-db.org/) and Cytoscape 3.7.2 software; (B) The enrichment analysis of CISD2 related genes in GO terms, including BP (biological process), CC (cell component) and MF (molecular function), and KEGG pathway.

**Additional file 2: Figure S2.** CISD2 expression endows the cellular resistance to ferroptosis inducer. (A–D) Analysis of the CISD2 expression by western blot in HT-1080 (A, C) or HL60 (B, D) cells, GAPDH was used as loading control; (E, F) Cell survival analysis in CISD2-modified HT-1080 cells after the treatment of Erastin (0–10 µM); (G, H) Cell survival analysis in CISD2-modified HL60 cells after the treatment of Erastin (0–15 µM); Cell survival in control cells without Erastin treatment was defined as 100%, the relative cell survival was calculated and graphed; ^★^*P* < 0.05 and ^★★^*P* < 0.01 between the indicated groups.

**Additional file 3: Figure S3.** Quantitative analysis of FTH expression in Fig. 3B.

**Additional file 4: Figure S4.** (A) Analysis of NQO1 expression by RT-PCR in CISD2 silenced HT-1080 cells with or without the treatment of erastin; (B) Quantitative analysis of NQO1 expression in Fig. 4A;^★^*P* < 0.05 and ^★★^*P* < 0.01 between the indicated groups.

## Data Availability

All data generated during this study are included either in this article or in Additional files.

## Abbreviations

DFO: Deferoxamine
Necro: Necrosuifonamide
NAC: *N*-acetylcysteine
PPI: Protein–protein interaction
RCD: Regulated cell death
HCC: Hepatocellular carcinoma
PDAC: Pancreatic ductal adenocarcinoma
NSCLC: Non-small-cell lung cancer
CISD2: CDGSH iron sulfur domain 2
Keap1: Kelch-like ECH-associated protein 1
NRF2: Nuclear factor erythroid 2-related factor 2
FTH: Ferritin heavy chain
HO-1: Heme oxygenase 1
GSH: Glutathione
ROS: Reactive oxygen species
