# Correction: Inhibition of CISD2 promotes ferroptosis through ferritinophagy-mediated ferritin turnover and regulation of p62–Keap1–NRF2 pathway

**DOI:** 10.1186/s11658-023-00478-1

**Published:** 2023-08-24

**Authors:** Yanchun Li, Bing Xu, Xueying Ren, Luyang Wang, Yaqing Xu, Yefeng Zhao, Chen Yang, Chen Yuan, Huanjuan Li, Xiangmin Tong, Ying Wang, Jing Du

**Affiliations:** 1 Department of Central Laboratory, Affiliated Hangzhou First People’s Hospital, Zhejiang University School of Medicine, Hangzhou, 310006 Zhejiang China; 2 Department of Clinical Laboratory, Laboratory Medicine Center, Zhejiang Provincial People’s Hospital (Affiliated People’s Hospital, Hangzhou Medical College), Hangzhou, 310014 Zhejiang China; 3 Department of Clinical Laboratory, Hangzhou Women’s Hospital, Hangzhou, 310016 Zhejiang China; 4 Department of Laboratory Medicine, The Second Affiliated Hospital of Zhejiang Chinese Medical University, Hangzhou, 310005 Zhejiang China

**Correction: Cellular & Molecular Biology Letters (2022) 27:81** **https://doi.org/10.1186/s11658-022-00383-z**

Following publication of the original article [1], the author informed us that they recently received a remark from a reader who pointed out a discrepancy in Fig. 5a. Specifically, the reader noted that the background in the second column appears to be different from the background in the other columns. Therefore, we providing an explanation for this observed difference. The order of presentation for the xenografts was reorganized. The data pertaining to the group of shCISD2 with Keap1 overexpression was not included in the final manuscript. Therefore, the background in the second column appears to be different. The original figure is presented as Additional file 1. Neither of these changes affects the results and conclusions of this study. The authors extend their apologies for any inconvenience caused.

### Supplementary Information

**Additional file 1.** The original figure of subcutaneous tumors in nude mice.
