# Supplementary figures and images for: Inhibition of CISD2 promotes ferroptosis through ferritinophagy-mediated ferritin turnover and regulation of p62–Keap1–NRF2 pathway
Source: Cell Mol Biol Lett. 2022 Sep 30;27:81. doi: 10.1186/s11658-022-00383-z (PMC9523958; doi:10.1186/s11658-022-00383-z)

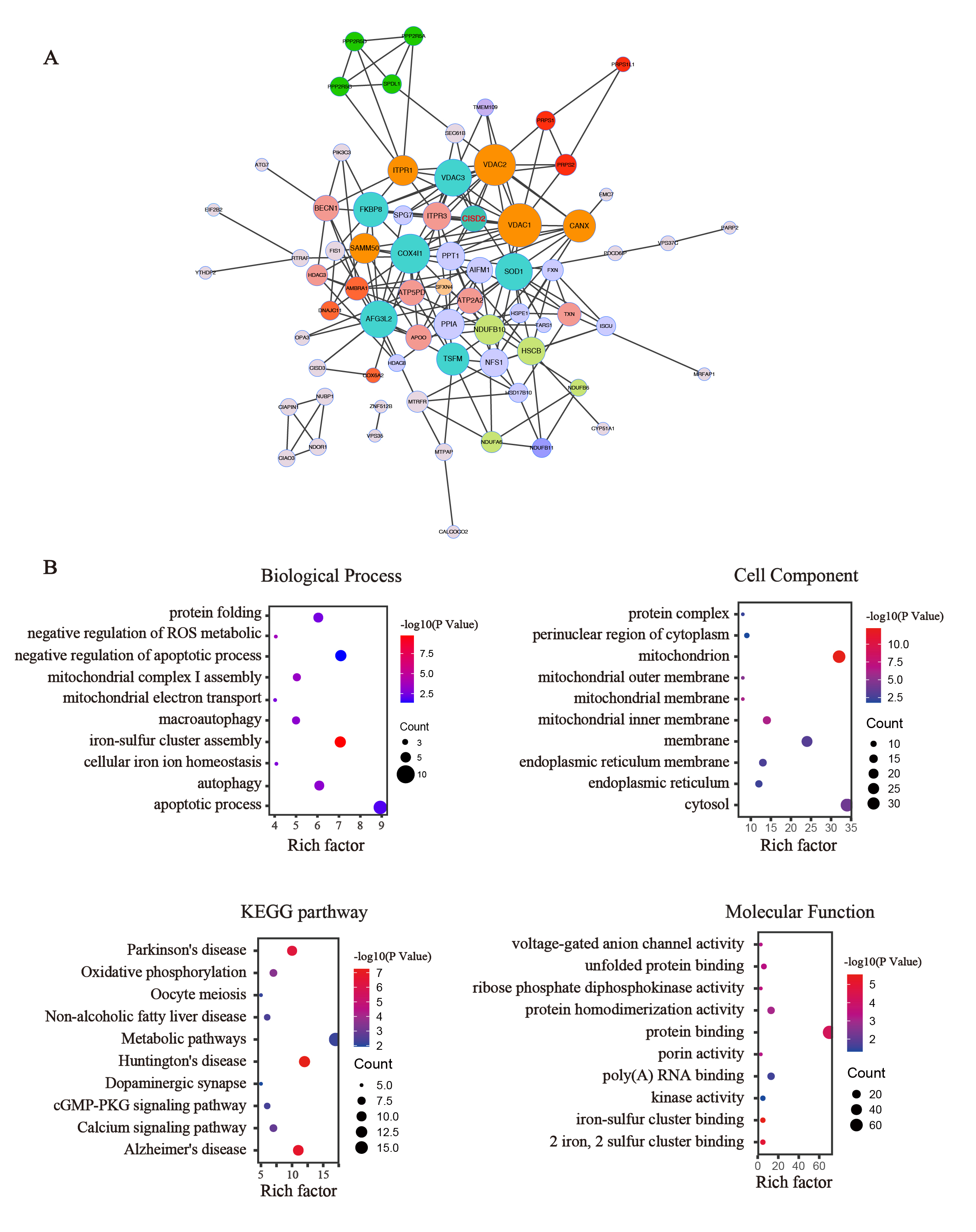

Supplement: Supplementary file 1 — Additional file 1: Figure S1. Enrichment analysis of CISD2 related genes. (A) Protein-protein interaction network of CISD2 from STRING website (https://www.string-db.org/) and Cytoscape 3.7.2 software; (B) The enrichment analysis of CISD2 related genes in GO terms, including BP (biological process), CC (cell component) and MF (molecular function), and KEGG pathway. [file 11658_2022_383_MOESM1_ESM.jpg]

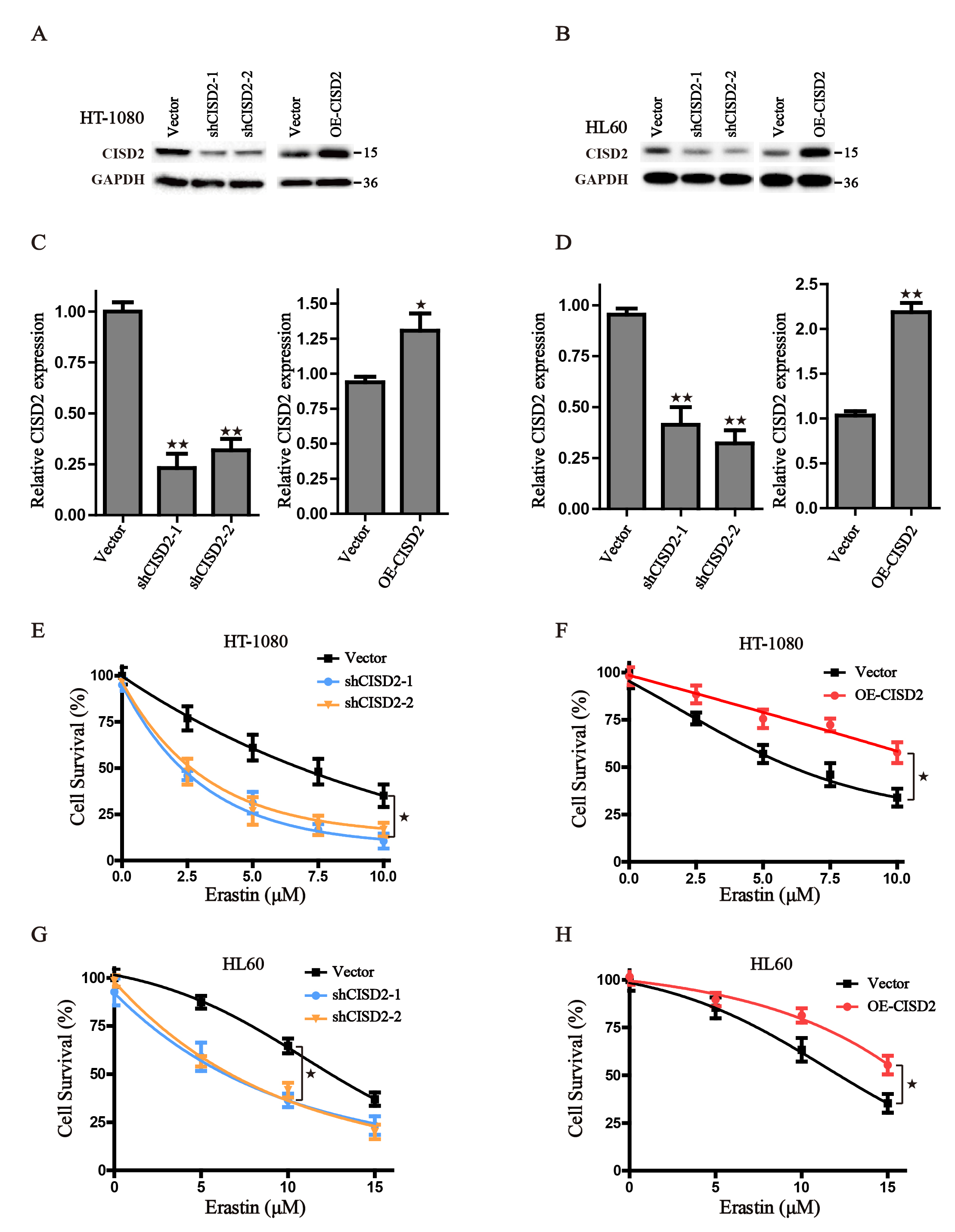

Supplement: Supplementary file 2 — Additional file 2: Figure S2. CISD2 expression endows the cellular resistance to ferroptosis inducer. (A–D) Analysis of the CISD2 expression by western blot in HT-1080 (A, C) or HL60 (B, D) cells, GAPDH was used as loading control; (E, F) Cell survival analysis in CISD2-modified HT-1080 cells after the treatment of Erastin (0–10 µM); (G, H) Cell survival analysis in CISD2-modified HL60 cells after the treatment of Erastin (0–15 µM); Cell survival in control cells without Erastin treatment was defined as 100%, the relative cell survival was calculated and graphed; ★P < 0.05 and ★★P < 0.01 between the indicated groups. [file 11658_2022_383_MOESM2_ESM.jpg]

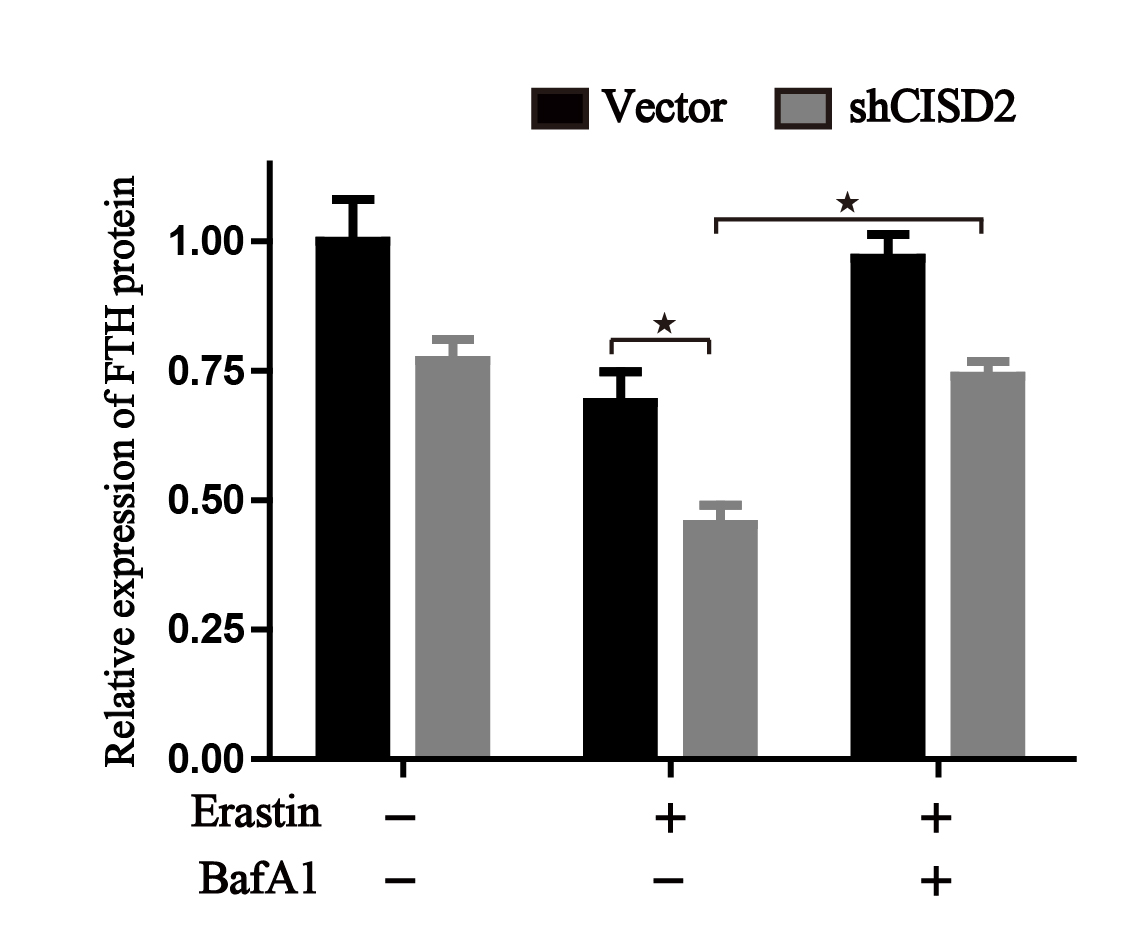

Supplement: Supplementary file 3 — Additional file 3: Figure S3. Quantitative analysis of FTH expression in Fig. 3B. [file 11658_2022_383_MOESM3_ESM.jpg]

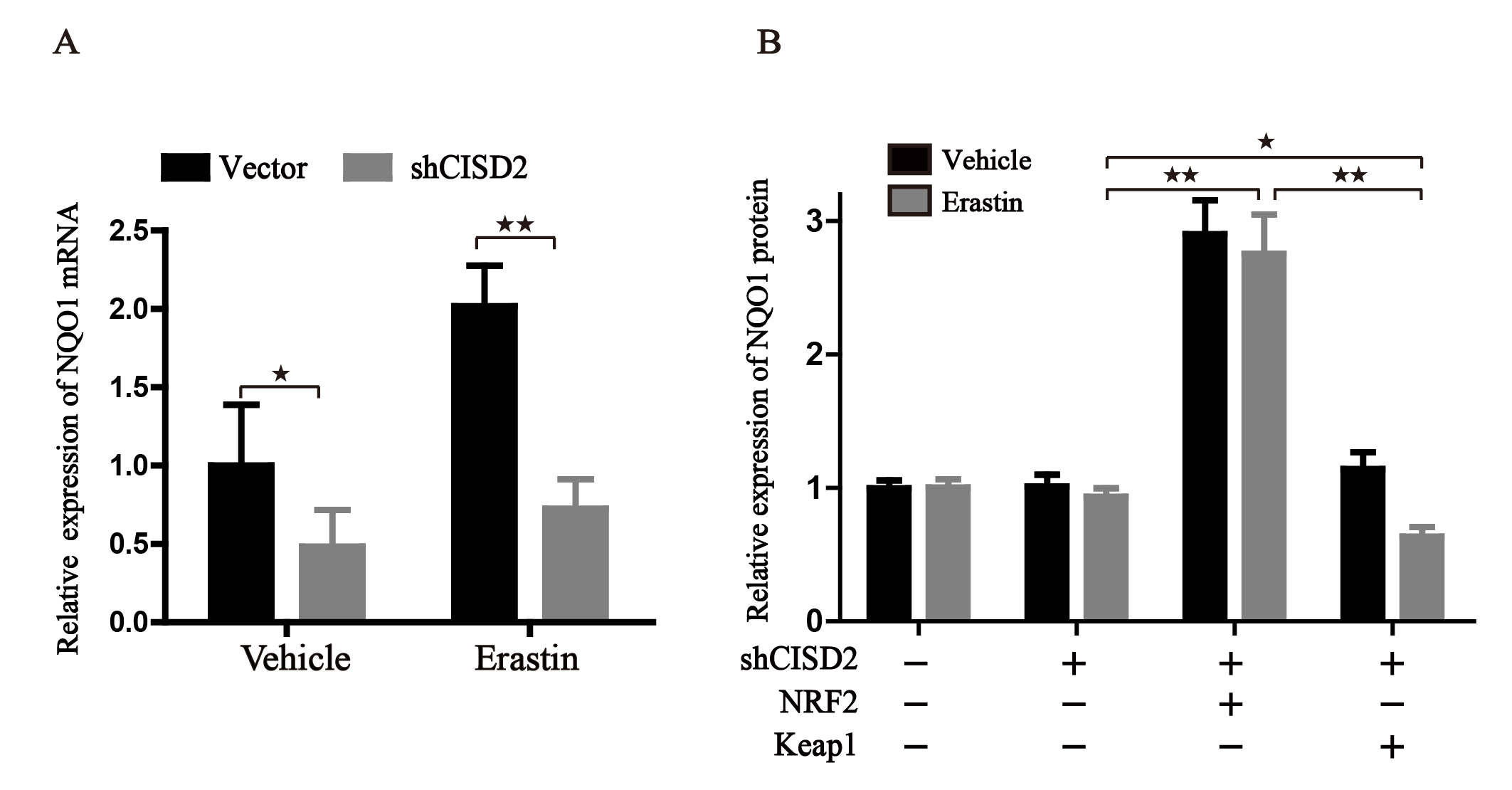

Supplement: Supplementary file 4 — Additional file 4: Figure S4. (A) Analysis of NQO1 expression by RT-PCR in CISD2 silenced HT-1080 cells with or without the treatment of erastin; (B) Quantitative analysis of NQO1 expression in Fig. 4A;★P < 0.05 and ★★P < 0.01 between the indicated groups. [file 11658_2022_383_MOESM4_ESM.jpg]
