# Supplementary figures and images for: Correction: Inhibition of CISD2 promotes ferroptosis through ferritinophagy-mediated ferritin turnover and regulation of p62–Keap1–NRF2 pathway
Source: Cell Mol Biol Lett. 2023 Aug 24;28:69. doi: 10.1186/s11658-023-00478-1 (PMC10464127; doi:10.1186/s11658-023-00478-1)

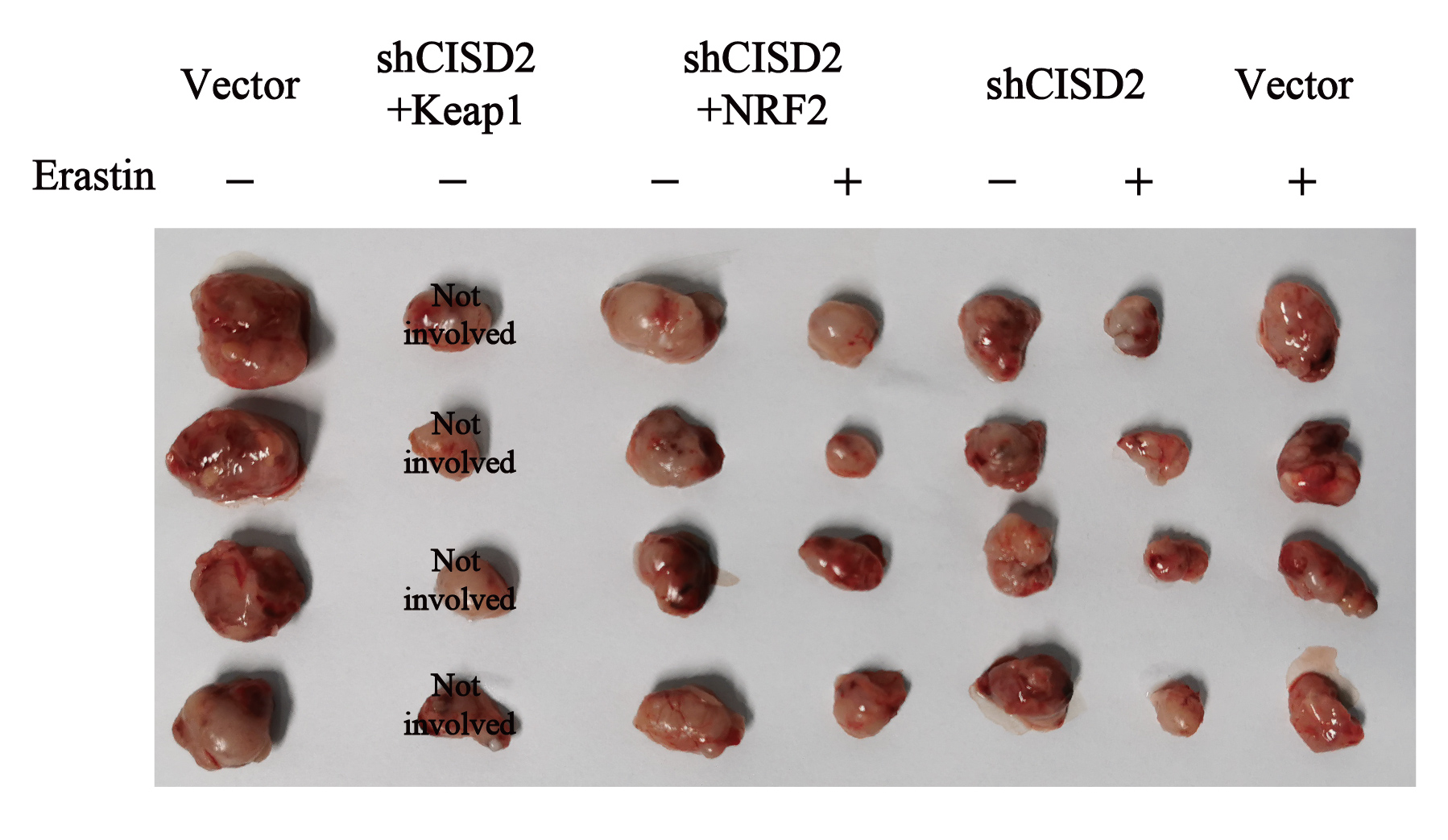

Supplement: Supplementary file 1 — Additional file 1. The original figure of subcutaneous tumors in nude mice. [file 11658_2023_478_MOESM1_ESM.jpg]
